# Semi-urgent pulmonary vein isolation using cryoballoon for haemodynamically unstable atrial fibrillation storm in a patient with low cardiac output syndrome: a case report

**DOI:** 10.1186/s12872-020-01682-z

**Published:** 2020-09-11

**Authors:** Toshiharu Koike, Fumiaki Mori, Ryozo Maeda, Ken Kobayashi, Masayuki Sakai, Kenjiro Oyabu, Yuko Matsui, Masafumi Yoshikawa, Kou Sugiyama, Yoichi Ajiro, Kazunori Iwade

**Affiliations:** Department of Cardiology, National Hospital Organization Yokohama Medical Center, 3-60-2 Harajuku, Totsuka-ku, Yokohama-shi, Kanagawa 245-8575 Japan

**Keywords:** Atrial fibrillation, Low cardiac output syndrome, Catheter ablation, Congestive heart failure, Pulmonary vein isolation, Cryoballoon ablation

## Abstract

**Background:**

Atrial fibrillation and heart failure are common coexisting conditions requiring hospitalisation for heart failure and death. Pulmonary vein isolation is a well-established option for symptomatic atrial fibrillation and for atrial fibrillation concomitant with heart failure with reduced left ventricular ejection fraction. Recently, pulmonary vein isolation using cryoballoon showed non-inferiority to radiofrequency ablation with respect to the treatment of patients with drug-refractory paroxysmal atrial fibrillation. However, the effectiveness of acute-phase rhythm control by semi-urgent pulmonary vein isolation using cryoballoon in patients with haemodynamically unstable atrial fibrillation storm accompanied with low cardiac output syndrome is unclear. Herein, we present a case in which semi-urgent pulmonary vein isolation using cryoballoon was effective for acute-phase rhythm control against drug-resistant and haemodynamically unstable repetitive atrial fibrillation tachycardia accompanied with low cardiac output syndrome.

**Case presentation:**

A 57-year-old man was hospitalised for New York Heart Association functional class 4 heart failure with atrial fibrillation tachycardia and reduced left ventricular ejection fraction of 20% accompanied with low cardiac output syndrome-induced liver damage. The haemodynamics collapsed during atrial fibrillation tachycardia, which had become resistant to intravenous amiodarone and repeated electrical cardioversions. In addition to atrial fibrillation, atrial tachycardia and common-type atrial flutter appeared on day 3. Multiple organ failure progressed gradually due to haemodynamically unstable atrial fibrillation tachycardia storm accompanied with low cardiac output syndrome. On day 4, to focus on treatment of heart failure and multiple organ failure, semi-urgent rescue pulmonary vein isolation using cryoballoon to atrial fibrillation and cavotricuspid isthmus ablation to common-type atrial flutter were performed for acute-phase rhythm control. Soon after the ablation procedure, atrial fibrillation and common-type atrial flutter were lessened, and sinus rhythm was restored. A stable haemodynamics was successfully achieved with the improvement of hepatorenal function. The patient was discharged on day 77 without complications.

**Conclusions:**

This case demonstrates that acute-phase rhythm control by semi-urgent pulmonary vein isolation using cryoballoon could be a treatment option in patients with haemodynamically unstable atrial fibrillation tachycardia storm accompanied with low cardiac output syndrome, which is refractory to cardioversion and drug therapy.

## Background

Atrial fibrillation (AF) and heart failure (HF) are common coexisting conditions with hospitalisation for HF and death [1]. Pulmonary vein isolation (PVI) is a well-established option for symptomatic paroxysmal AF [2] and for AF concomitant with HF with reduced left ventricular ejection fraction (HFrEF) [3–6]. PVI for patients with AF concomitant with HFrEF demonstrated significant reduction in overall mortality rate and incidence of hospitalisation for worsening HF and improvement in left ventricular ejection fraction (LVEF) compared with the conventional drug therapy [5]. The cryoballoon is a recently developing ablation tool that showed non-inferior efficacy and overall safety [7–9]. However, the role and significance of acute-phase rhythm control by semi-urgent PVI is not yet established. Herein, we present a case in which semi-urgent PVI using cryoballoon was effective for acute-phase rhythm control against drug-resistant and haemodynamically unstable repetitive AF tachycardia storm accompanied with low cardiac output syndrome (LOS) and LOS-induced multiple organ failure (MOF).

## Case presentation

A 57-year-old man was referred to our hospital for New York Heart Association functional class 4 HF and extremely elevated liver enzymes. He had palpitation from 2 weeks ago which was accompanied with orthopnoea and serious fatigue on admission, despite being healthy without any history of AF. The electrocardiography showed AF tachycardia of approximately 180 beats per minute, and the bedside echocardiography showed low LVEF of 20%. In addition to fatigue, coexisting hypotension and elevated lactate of 13 mmol/L indicated LOS. Electrical cardioversion was conducted, barely terminated AF successfully on day 1, and restored blood pressure and urinalysis response to intravenous furosemide. Intravenous landiolol hydrochloride was administered for AF tachycardia. HF and LOS were treated with intravenous dobutamine, intravenous furosemide and oral tolvaptan, and non-invasive positive pressure ventilation under mild sedation using intravenous dexmedetomidine hydrochloride. On day 2, torsade de pointes suddenly occurred subsequent to a premature atrial beat in a long-short manner of coupling interval with QT prolongation. Cardiopulmonary resuscitation with electric cardioversions, intratracheal intubation, and establishment of mechanical ventilation were carried out, which achieved return of spontaneous circulation. Temporary atrium-atrium inhibited pacing was emergently established to shorten prolonged QT and maintain regular heart rate. However, AF tachycardia recurred repetitively. Repeated electrical cardioversions failed to terminate AF. Hypotension continued along with oliguria. The liver dysfunction was further exacerbated with aspartate aminotransferase of 11,708 U/L. The intravenous amiodarone was started. On day 3, AF tachycardia with hypotension still occurred despite intravenous amiodarone and gradually became resistant to electrical cardioversions. The atrial tachycardia (AT) and common-type atrial flutter (AFL) appeared in addition to AF. On day 4, disseminated intravascular coagulation (DIC) was diagnosed according to the Japanese Society on Thrombosis and Hemostasis criteria, with DIC score of 6 points [10]. Acute kidney injury was diagnosed according to the Kidney Disease Improving Global Outcomes criteria, fulfilling both serum creatine and urine output criteria [11]. At this time, because the patient’s general condition was becoming worse and more resistant to treatment, we decided to perform semi-urgent rescue ablation to AF and common-type AFL for acute-phase rhythm control to treat HF and MOF.

The patient’s family agreed with our treatment policy and signed informed consent for the semi-urgent rescue ablation procedure.

**Fig. 1:**
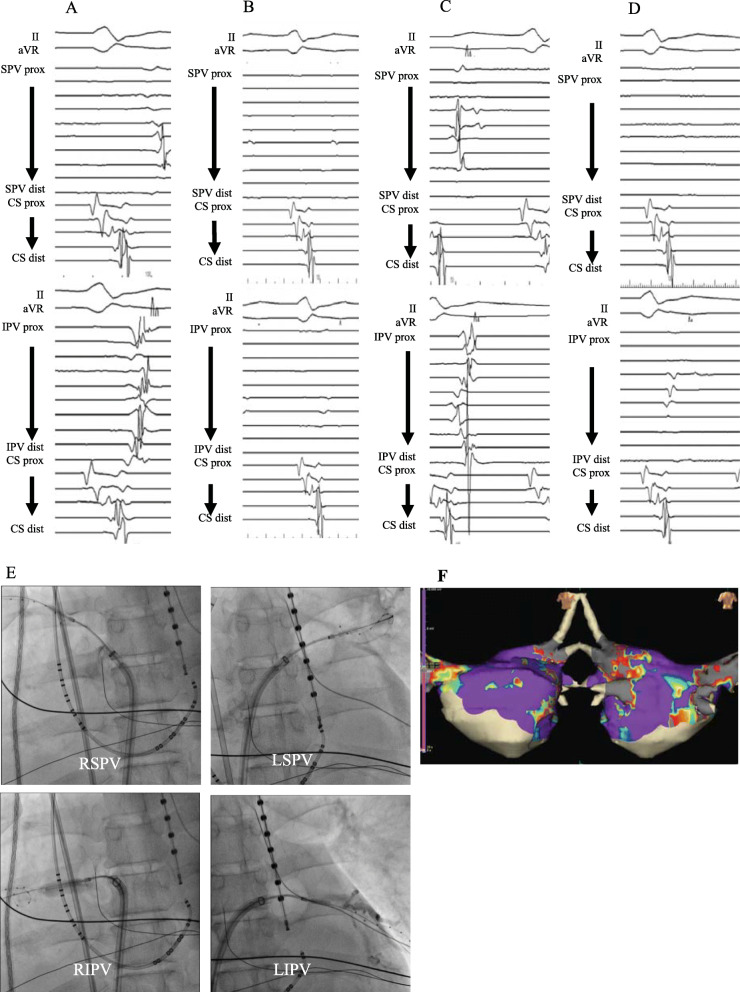
Pulmonary vein isolation using cryoballoon. **a:** Baseline intracardiac electrograms of the left pulmonary veins before isolation. **b:** Intracardiac electrograms after the left pulmonary vein isolation. **c:** Baseline intracardiac electrograms of the right pulmonary veins before isolation. **d:** Intracardiac electrograms after the right pulmonary vein isolation. **e:** Fluoroscopic AP image demonstrating positions of the cryoballoon for all pulmonary veins. **f:** Three-dimensional map with grey area representing ablated area by pulmonary vein isolation using cryoballoon. CS, coronary sinus; dist, distal bipole; prox, proximal bipole; IPV, inferior pulmonary vein; LIPV, left inferior pulmonary vein; LSPV, left superior pulmonary vein; RIPV, right inferior pulmonary vein; RSPV, right superior pulmonary vein; SPV, superior pulmonary vein

On the same day, prior to the procedure, trans-oesophageal echocardiography was performed and found no detectable thrombus in the left atrium (LA). PVI to the AF and cavotricuspid isthmus ablation to common-type AFL were planned. Fortunately, the patient’s haemodynamics did not collapse at the time of ablation, we performed our institutional standard procedure including 3-D mapping. The cryoballoon was chosen as a PVI catheter because cryoballoon ablation was expected to have a shorter procedure time and lesser thrombogenic effect than other catheter types [7, 12]. A 20-polar catheter (Response™; Abbott, St. Paul, MN, USA) was placed in the coronary sinus. A three-dimensional electroanatomical mapping was constituted by a cardiac mapping system (EnSite velocity™; Abbott). Transseptal access was obtained using the standard Brockenbrough needle technique with intracardiac ultrasound and fluoroscopic guidance, and an 8-Fr SL0 sheath (Swartz™; Abbott) was inserted into the LA. The cardiac geometry including all pulmonary veins (PV) was established using a 20-pole circular mapping catheter (Reflexion Spiral™; Abbott). No left atriography was conducted because of acute kidney injury. Then, we changed the SL0 sheath to a steerable sheath (FlexCath Advance™; Medtronic Inc., Minneapolis, MN, USA) and inserted a 28-mm second-generation cryoballoon catheter (Arctic Front Advance™; Medtronic Inc.). In each PV, the cryoballoon was placed at the ostium of each PV in turns, and cryoballoon ablation was performed after complete occlusion of each PV as confirmed by the minimum amount of contrast agent. During cryoenergy deliveries, the oesophageal temperature and diaphragmatic compound motor action potential were monitored to avoid LA-oesophageal fistula and phrenic nerve injury. Additional touch-up radiofrequency ablation using FlexAbility™ (Abbott) to the residual LA-PV conduction gap at the bottom of the right inferior PV after cryoballoon ablation was conducted, and complete PVI was achieved (detailed PVI procedural data is shown in Table 1 and Fig. 1). Subsequently, a 20-polar catheter (Livewire™; Abbott) was placed around the tricuspid annulus, confirming that the AFL was cavotricuspid-isthmus dependent. The cavotricuspid isthmus ablation for common-type AFL was performed by standard procedure using radiofrequency ablation catheter and successfully achieved bidirectional block. The whole procedure was finished uneventfully and restored sinus rhythm and blood pressure of approximately 110 mmHg.

**Table 1:**
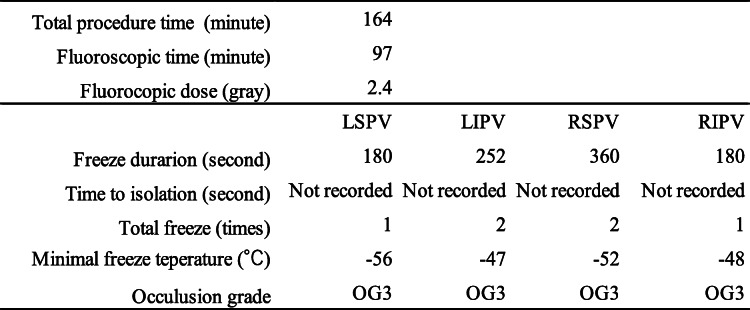
The procedural data of pulmonary vein isolation using cryoballoon

The occlusion grade (OG) was scored as follows: OG3 (complete occlusion), OG2 (incomplete occlusion with slight leakage), OG1 (poor occlusion with massive leakage)

*LSPV* left superior pulmonary vein, *LIPV* left inferior pulmonary vein, *RSPV* right superior pulmonary vein, *RIPV* right inferior pulmonary vein, *OG* Occlusion grade

**Fig. 2:**
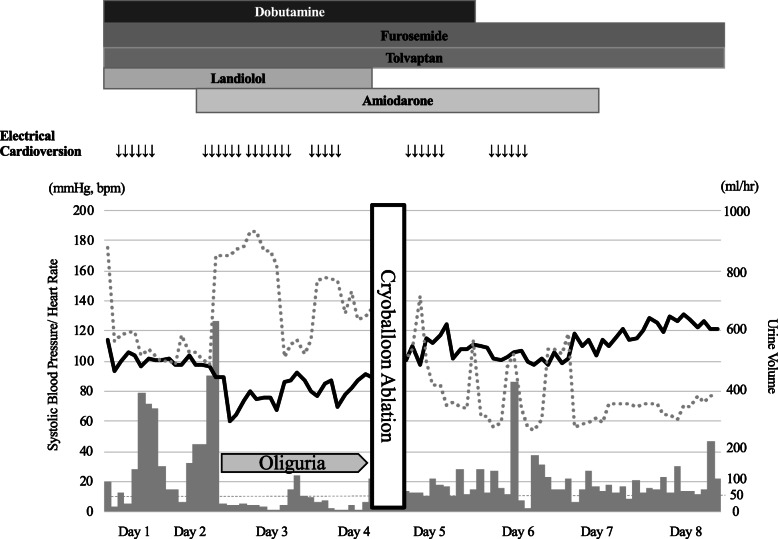
Acute-phase clinical profile. Atrial fibrillation tachycardia accompanied with hypotension and oliguria. Semi-urgent pulmonary vein isolation using cryoballoon improved haemodynamics. Solid arrows indicate electrical cardioversions. Bar graph represents urine volume per hour. Solid line graph represents systolic blood pressure. Dotted line graph represents heart rate. BP, blood pressure

Thereafter, AF and other atrial arrhythmias seldom occurred and was terminated by single electrical cardioversion (Fig. 2). Normal blood pressure and urine output were restored (Fig. 2). The hepatic and renal functions were improved gradually as well. On day 7, intravenous amiodarone was discontinued. On day 11, the patient was weaned from the ventilator support. The DIC was restored eventually. On day 18, pulmonary abscess that required long-term antimicrobial treatment was cured. On day 22, AF recurred, but oral amiodarone was restarted and suppressed the AF. Although the patient had a pulmonary abscess that required approximately one-month antimicrobial treatment after ventilator withdrawal, he was discharged alive on day 72. The elective coronary angiography and left ventriculography revealed no significant coronary stenosis and LVEF normalisation, diagnosing that the tachycardia-induced cardiomyopathy due to AF tachycardia was the cause of reduced left ventricular function. For 2 years, albeit the discontinuation of HF drugs and amiodarone, the patient has been free from HF symptoms and atrial arrhythmias including AF and AFL.

## Discussion and conclusion

To our knowledge, this is the first report of semi-urgent rescue PVI using cryoballoon for acute-phase rhythm control against amiodarone-resistant AF tachycardia storm causing LOS and LOS-induced MOF with DIC in a tachycardia-induced cardiomyopathy.

AF may cause adverse haemodynamic effects and lead to decrease in cardiac output through the loss of atrial contraction, reduction of left ventricular filling due to rapid ventricular rates and irregular RR interval, increase maximal oxygen consumption, and exacerbate mitral and tricuspid regurgitation [1]. Therefore, restoration of sinus rhythm in AF patients can be expected to improve cardiac output and decrease maximal oxygen consumption [3, 4]. PVI is the established treatment for the rhythm control strategy of AF even refractory to antiarrhythmic drugs [2] because PVI has an anti-AF mechanism different from that of drugs, such as eliminating AF substrate, denervating the autonomic nerve, and most importantly eliminating AF triggers arising from PVs [13, 14]. On the contrary, the role and significance of acute-phase rhythm control by semi-urgent PVI is not yet established. However, Morishima et al. reported a similar case. They described that the semi-urgent rescue PVI could eliminate haemodynamically unstable AF storm and contribute to the improvement of haemodynamic states in a patient with an acute myocardial infarction, although the patient died from ventricular fibrillation as a complication of acute myocardial infarction [15]. This report also supported the benefit of semi-urgent rescue PVI on acute-phase rhythm control against haemodynamically unstable AF tachycardia. In addition, the present case had LOS-induced MOF and DIC in which organ perfusion flow was originally reduced due to left ventricular dysfunction and was additionally reduced by AF tachycardia storm [1]. Furthermore, especially in LOS-induced shock states, the importance of cardiac output increase and the resultant increase in blood pressure by rhythm control might be emphasised because of the following pathophysiology: (1) central shift of the circulating blood and organ perfusion in contrast flow reduction due to neurohormonal response [16], (2) change of the source of liver blood perfusion supply from the portal vein to the hepatic artery due to hepatic arterial buffer reaction [17], and (3) dependency of renal blood perfusion on cardiac output and on blood pressure in hypotension [18]. Together with these considerations, we thought that the acute-phase rhythm control by semi-urgent PVI would have a certain role in LOS-induced MOF due to AF tachycardia, at least in elimination as aggravation factor, and would enable focus on the intensive care of original diseases and disorders.

We chose the cryoballoon ablation for semi-urgent rescue PVI in the present case. Cryoballoon ablation is a balloon-based ablation system using cryoenergy. In recent years, cryoballoon ablation has become the most effective alternative approach to radiofrequency catheter ablation showing non-inferiority to radiofrequency catheter ablation in freedom from AF/AT recurrence and overall safety [7–9]. Several clinical studies showed shorter procedure time in cryoballoon ablation than that in radiofrequency catheter ablation [7, 9]. An animal study showed that cryoballoon ablation has lower incidence of thrombus formation than radiofrequency catheter ablation [12]. The shorter procedure time may suppose lesser load in intensive care setting. The lower incidence of thrombus formation may be favourable in intensive care setting accompanied with DIC. Although the significance of rescue cryoballoon ablation is unknown, we considered that cryoballoon ablation could be a favourable tool when we were required to perform semi-urgent rescue PVI, like in the present case. On the contrary, balloon-based ablation including cryoballoon ablation generally uses additional amount of contrast medium. We might consider performing the balloon-based ablation including cryoballoon ablation without left atriography for the patient with acute kidney injury, like in the present case. At the same time, we would like to emphasise the importance of careful and timely assessment of the benefit and risk of semi-urgent rescue PVI using cryoballoon because semi-urgent PVI using cryoballoon could be a complex procedure for a complex case.

In conclusion, acute-phase rhythm control by semi-urgent PVI using cryoballoon might be a considerable treatment option in patients with haemodynamically unstable AF tachycardia which is refractory to cardioversion and drug therapy and accompanied with LOS and LOS-induced MOF with DIC.

## Data Availability

The datasets used and/or analysed during the current study are available from the corresponding author upon reasonable request.
